# When SUV Matters: FDG PET/CT at Baseline Correlates with Survival in Soft Tissue and Ewing Sarcoma

**DOI:** 10.3390/life11090869

**Published:** 2021-08-24

**Authors:** Ruben I. Hack, Anton S. Becker, Beata Bode-Lesniewska, G. Ulrich Exner, Daniel A. Müller, Daniela A. Ferraro, Geoffrey I. Warnock, Irene A. Burger, Christian Britschgi

**Affiliations:** 1 Department of Nuclear Medicine, University Hospital Zürich, University of Zürich, 8091 Zürich, Switzerland; ruben.hack@spitaluster.ch (R.I.H.); Daniela.Ferraro@usz.ch (D.A.F.); 2 Department of Interventional and Diagnostic Radiology, University Hospital Zürich, University of Zürich, 8091 Zürich, Switzerland; beckera1@mskcc.org; 3 Institute of Pathology and Molecular Pathology, University Hospital Zürich, University of Zürich, 8091 Zürich, Switzerland; beata.bode@patho.ch; 4 Orthopaedie Zentrum Zuerich, 8038 Zürich, Switzerland; guexner@gmail.com; 5 Balgrist University Hospital Zürich, Forchstrasse 340, 8008 Zürich, Switzerland; daniel.mueller@balgrist.ch; 6 PMOD Technologies LLC, 8091 Zürich, Switzerland; GeoffreyIain.Warnock@usz.ch; 7 Department of Nuclear Medicine, Kantonsspital Baden, 5404 Baden, Switzerland; 8 Department of Medical Oncology and Hematology, University Hospital Zürich, University of Zürich, 8091 Zürich, Switzerland; Christian.Britschgi@usz.ch

**Keywords:** staging, sarcoma, survival, PET/CT, SUV, TLG

## Abstract

Introduction: The role of positron-emission tomography/computed-tomography (PET/CT) in the management of sarcomas and as a prognostic tool has been studied. However, it remains unclear which metric is the most useful. We aimed to investigate if volume-based PET metrics (Tumor volume (TV) and total lesions glycolysis (TLG)) are superior to maximal standardized uptake value (SUVmax) and other metrics in predicting survival of patients with soft tissue and bone sarcomas. Materials and Methods: In this retrospective cohort study, we screened over 52′000 PET/CT scans to identify patients diagnosed with either soft tissue, bone or Ewing sarcoma and had a staging scan at our institution before initial therapy. We used a Wilcoxon signed-rank to assess which PET/CT metric was associated with survival in different patient subgroups. Receiver-Operating-Characteristic curve analysis was used to calculate cutoff values. Results: We identified a total of 88 patients with soft tissue (51), bone (26) or Ewing (11) sarcoma. Median age at presentation was 40 years (Range: 9–86 years). High SUVmax was most significantly associated with short survival (defined as <24 months) in soft tissue sarcoma (with a median and range of SUVmax 12.5 (8.8–16.0) in short (n = 18) and 5.5 (3.3–7.2) in long survival (≥24 months) (n = 31), with (*p* = 0.001). Similar results were seen in Ewing sarcoma (with a median and range of SUVmax 12.1 (7.6–14.7) in short (n = 6) and 3.7 (3.5–5.5) in long survival (n = 5), with (*p* = 0.017). However, no PET-specific metric but tumor-volume was significantly associated (*p* = 0.035) with survival in primary bone sarcomas (with a median and range of 217 cm^3^ (186–349) in short survival (n = 4) and 60 cm^3^ (22–104) in long survival (n = 19), with (*p* = 0.035). TLG was significantly inversely associated with long survival only in Ewing sarcoma (*p* = 0.03). Discussion: Our analysis shows that the outcome of soft tissue, bone and Ewing sarcomas is associated with different PET/CT metrics. We could not confirm the previously suggested superiority of volume-based metrics in soft tissue sarcomas, for which we found SUVmax to remain the best prognostic factor. However, bone sarcomas should probably be evaluated with tumor volume rather than FDG PET activity.

## 1. Introduction

Sarcomas are a very rare and heterogeneous group of tumors of various histological subtypes, arising in soft tissue or bone. Differences in tumor biology result in a wide range of clinical behavior from dismal prognosis to long-term survival even within one histological subtype. However, current tools to predict survival are limited. Apart from histological grading, there are other prognostic factors to determine how aggressive a tumor is. In soft tissue sarcomas (STS), for instance, it is the tumor location, the size of the primary tumor and its grade [1,2]. Deeper and more proximally located sarcomas have a worse prognosis than superficially located sarcomas of the distal extremities, for instance. As for bone sarcomas (BS) also in osteosarcoma, initial tumor size has been shown to be a prognostic factor and to predict presence of metastases [3]. For BS and ES, in general, tumor volume is an important factor for staging and has an impact on prognosis; other negative prognostic factors include the presence of a pathological fracture and elevated serum levels of alkaline phosphatase (AP) and lactate dehydrogenase (LDH) [4].

Radiologic examinations of all sorts can potentially provide both the size and location of a tumor. MRI scans have been used early in the assessment of STS, not only for measurement purposes, but also to gain information about tumor metabolism via spectroscopy [5]. Further, the absence of a peritumoral edema in MRI scans has been described as a potentially positive prognostic factor [6]. In Ewing sarcoma family of tumors (ESFT), larger tumor volume in MRI scans has been associated with poorer survival [7].

The role of positron-emission tomography/computed-tomography (PET/CT) scans using ^18^F-Fluorodeoxyglucose (FDG) in the management of sarcomas has been primarily to rule out metastatic disease. However, it has also been discussed as a potential prognostic tool [8]. In cohorts of different STS the maximum standardized uptake value (SUV_max_) was reported to correlate with overall survival (OS) [9,10], but also in more specific histological subgroups, such as synovial sarcoma [11]. Later, also volume-based PET metrics such as total lesion glycolysis (TLG) were suggested to be superior to predict outcome in STS, as they provide more information on tumor metabolism than a single voxel maximum value [12,13]. Others explicitly concluded that volume-based PET metrics do not add any additional information on survival prediction in STS [10]. In a meta-analysis, SUV_max_, metabolic tumor volume (MTV) and TLG were all found to be prognostic in STS [14]. Also in osteosarcomas SUV_max_ was found to be a prognostic factor by some [15], while Byun et al. suggested that MTV might be a better predictor for survival than SUV_max_ in osteosarcomas [16]. Other studies investigating mixed cohorts of bone sarcomas (including mostly osteosarcoma and some ESFT) found no correlation between survival and SUV_max_ [9]. In ESFT, SUV_max_ was shown to correlate with OS and progression free survival (PFS) [17,18,19].

Assessing the aggressiveness of the disease is essential in the management of sarcoma patients to guarantee treatment adequate to the disease and in the case of STS to help decide which patients might be candidates for adjuvant or neo-adjuvant chemotherapy. This is done by integrating histology, grade and anatomical location in relation to the fascia (deep versus superficial) and tumor dimensions into the clinical decision-making process for example in STS [20]. This approach has limitations: especially tumor heterogeneity can lead to a sampling error while performing the initial biopsy to establish the histopathological diagnosis. Radiology and nuclear medicine provide tools to assess a tumor comprehensively and non-invasively. However, the existing data are inconclusive and rather contradictory about which PET metric provides the best prognostic factor for which group of sarcomas.

We aimed to investigate if volume derived from the CT scan, or a volume-based PET metric (TLG) are superior compared to SUV_max_ in association with outcome in different types of sarcomas and to determine, if there are cutoff values that best predict outcome for different histological subtypes.

## 2. Materials and Methods

### 2.1. Patient Selection

In this retrospective study, all FDG-PET/CT scans performed at the University Hospital Zurich between 2001–2014 were identified. Using the search terms for different sarcoma subtypes and the respective ICD-10 codes for sarcomas, all reports were automatically and subsequently manually searched for initial staging examinations of sarcomas. In accordance with the approval of the local ethics committee (BASEC Nr. 2017-00475), informed consent was waived. Patients whose initial scans were performed after surgery, radiotherapy or chemotherapy were excluded. Tumor biopsy was not considered surgery. Interpretation of sarcoma histology is a challenging task and a diagnosis can vary among different pathologists. Furthermore, the classification of sarcomas and the nomenclature of histological subtypes has changed over the years. Whenever possible, the sarcoma diagnosis was therefore reviewed and validated by our specialized reference pathologist (BB) to verify the exact histopathological diagnosis according to the currently valid “*WHO Classification of Tumours of Soft Tissue and Bone (2013)*” [21]. For the analysis, we split the patients in three groups: STS (comprising non uterine leiomyosarcomas, liposarcomas, angiosarcomas, rhabdomyosarcomas, undifferentiated pleomorphic sarcomas, synovial sarcomas, myxofibrosarcomas and others); BS (comprising osteosarcomas, chondrosarcomas, undifferentiated pleomorphic sarcomas of the bone and leiomyosarcomas of the bone), and ESFT (Ewing sarcomas and other Ewing sarcoma family tumors). Clinical data of the patients were collected from the patients’ records for survival analyses.

### 2.2. PET/CT Scans

All patients were scanned on a dedicated PET/CT machine (GE Healthcare DSTX, 16-or 64-slices CT, 7–8 frames, frame time 1.5 or 2 min). Fasting for 6 h prior to the study was mandatory. Patients were not allowed to consume sweetened beverages and chewing gum. Blood glucose was measured prior to the FDG-injection and had to be below 120 mg/dl. Patients received 2–4 MBq ^18^F-FDG per kilogram bodyweight, followed by a 45–60 min uptake period. Afterwards, a low-dose, attenuation correction CT scan was acquired (100–120 kV, approx. 80 mA), followed by the PET scan from mid-thigh to the vertex of the skull followed by a scan of the lower extremities depending on tumor localization.

### 2.3. Image Analysis

The PET/CT data of the included patients were all processed using the *PMOD Technologies LLC software* (www.pmod.com, accessed on 30 April 2018), and the primary tumors were manually outlined on all slices with care, thus obtaining accurate volumes of interest (VOI) representing the radiologic tumor volumes. All VOIs were created by the same reader (RIH) ensuring a consistent interpretation of tumor volumes under the supervision of a nuclear radiologist (IAB). The VOI were used on the PET data, as well as on the CT data. The volume outlined in the CT scan was always checked to fit the PET-scan best and minor changes on the basis of FDG-avidity could be made. Decay corrected activity on PET images was normalized using the following formula to obtain standardized uptake value (SUV): $\mathrm{SUV}=\frac{\mathrm{Mean}\;\mathrm{VOI}\;\mathrm{activity}\;\mathrm{concentration}\;\left(\frac{\mathrm{kBq}}{\mathrm{mL}}\right)}{\mathrm{Injected}\;\mathrm{dose}\;\left(\mathrm{MBq}\right)/\mathrm{Body}\;\mathrm{weight}\;\left(\mathrm{kg}\right))}$.

**Figure 1:**
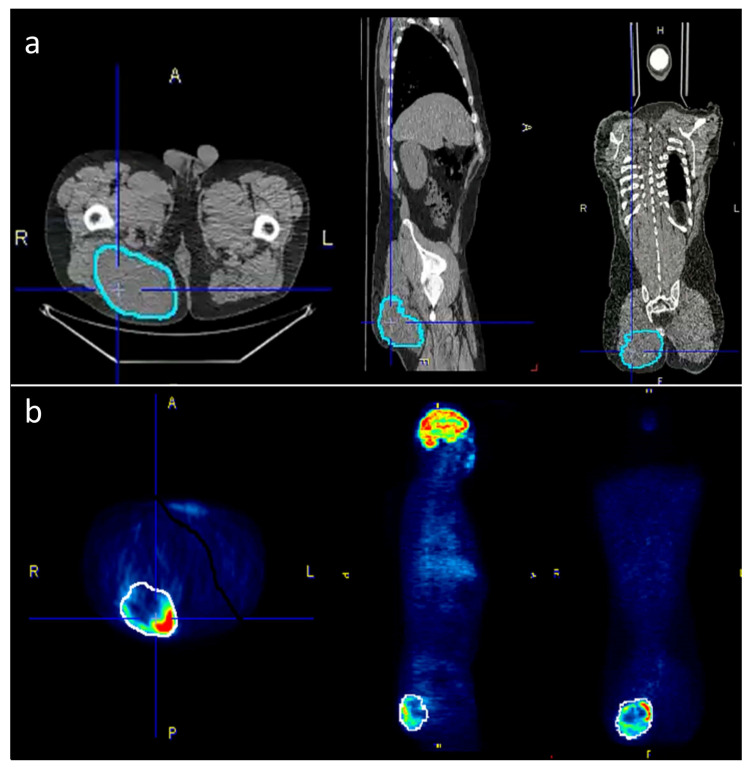
To accurately outline the tumors, the VOIs were selected semi-automatically on the PET-images where possible and then manually adjusted to the tumor borders on the CT scan. (**a**) The corrected VOIs were then copied back to the PET-scan (**b**) in order to obtain identical VOIs for both PET and CT data.

Within the pre-defined volume based on the CT data, the following PET/CT metrics were calculated and exported: SUV_max_, SUV_avg_, SUV_median_, SUV_min_ and TLG (defined as SUV_avg_ * tumor volume). For CT-parameters, the tumor volume and the Hounsfield units (HU) were acquired within the same VOI. Figure 1 is giving an example for tumor segmentation in a patient with a large gluteal STS.

### 2.4. Statistics

For the statistical analysis, we divided the patients into a long and short survival group. Survival time was defined as the time since the initial PET scan until death or last follow-up. The threshold for long survival was defined as 24 months or more. Short survival was defined as less than 24 months. Patients who did not die of their disease but were lost to follow-up in this time frame (n = 5, one of which died of a reason unrelated to the sarcoma) were not considered for the survival analysis, leaving 83 patients. Statistical calculations were performed using *IBM SPSS Statistics 24.* We used a Wilcoxon signed-rank test to analyze, which PET/CT metrics were associated with survival in the different patient subgroups. A Spearman’s rho coefficient was used to correlate survival time with these PET metrics. A *p*-value < 0.05 was considered significant. In case of significant association, receiver-operating-characteristic (ROC) curves were used to determine the area under the curve (AUC) in each group, which allowed to use cutoff values using the Youden index. Only patients with at least 24 months of follow-up or patients who died of their disease within this time frame (n = 83) were considered for this analysis. For Kaplan–Meier estimation of survival curves, all 88 patients were included. Patients were grouped into one of the three categories mentioned above (STS, BS and ESFT). A statistical analysis for the individual histologies was not possible in many cases due to low patient numbers. However, whenever possible we investigated these subgroups as well.

## 3. Results

### 3.1. Patient Cohort

**Figure 2:**
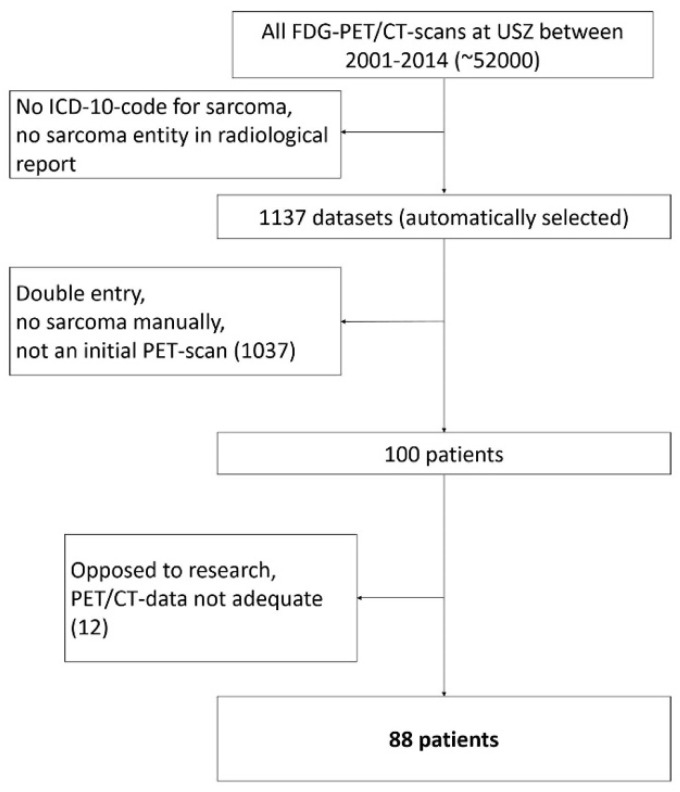
Patient selection flow chart.

The screening of all PET/CT scans resulted in a total of n = 88 eligible patients (Figure 2). Of the 88 patients 49 were male and 39 female. Median age at presentation was 40 years (Range: 9–86 years). At the time the initial scan was performed, 70 patients (36 men and 34 women) had localized disease and 18 patients (13 men and 5 women) had metastases on PET/CT scan. In the initial scan, a mean dose of 347 (±51) MBq was administered. Median Follow-up time was 47 months (Range 1–157). At the end of follow-up, 48 patients were alive and 35 free of disease. Follow-up ended when a patient died or at the last point in time he or she was reportedly alive. The patient characteristics are summarized in Table 1.

The exact histopathological diagnosis was determined for every patient. Eighty of 88 histopathology samples were reevaluated, resulting in 31 different diagnostic subcategories as shown in Table 2. For further analysis, these categories were summarized in the three aforementioned main groups: bone sarcoma (BS), soft tissue sarcoma (STS) and Ewing sarcoma family tumors (ESFT).

### 3.2. PET Metrics for Different Subgroups

PET quantification metrics between the three subgroups are shown in Table 3. There was a highly significant difference in the average Hounsfield density, which is not surprising since we compared tumors arising mostly in bone (BS, ESFT) with STS. In addition, TLG was significantly different between the three groups with BS showing the lowest median TLG value.

### 3.3. PET Metrics Predicting Survival

**Figure 3:**
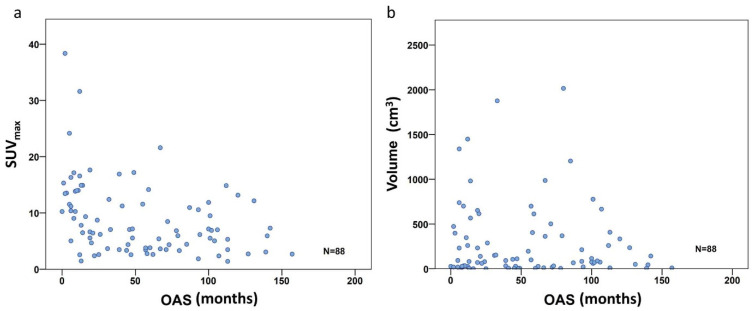
Correlation scatterplot for SUV_max_ (**a**) and volume (**b**) including all 88 patients. Only the SUV_max_ group shows significant correlation with OS (r = −0.414, *p* < 0.001), volume did not (r = −0.042, *p* = 0.699).

**Figure 4:**
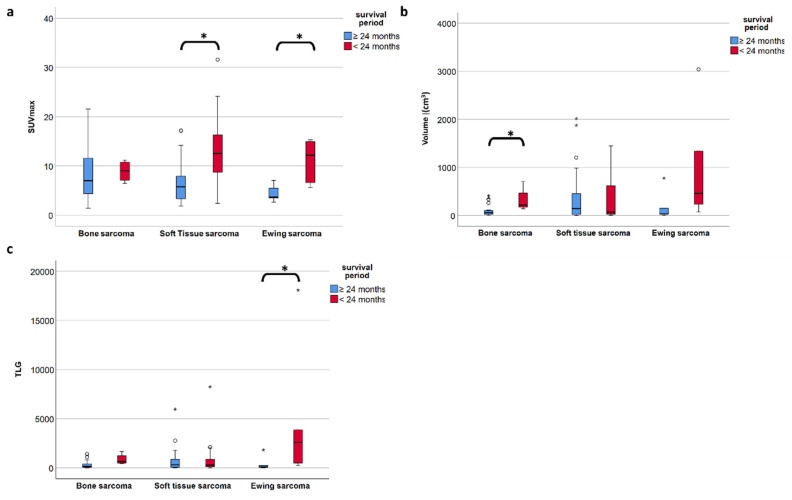
(**a**–**c**) The median SUV_max_ is significantly different in short- and long-term survival patients in soft tissue and Ewing sarcoma but not in bone sarcoma (**a**), whereas in bone sarcoma is the volume of the primary tumor is significantly different in short vs. long term survival (**b**). TLG only proves significant in ESFT but less so than SUV_max_. (**c**). A p-value less than 0.05 is considered significant (*).

To investigate the PET metric most strongly associated with long-term survival (which we defined as more than 24 months after diagnosis), we included the 83 patients with follow-up of at least or death within 24 months. In the entire study population comprising all three subgroups, the FDG uptake represented by SUV_max_ did show a significant negative correlation with overall survival time according to Spearman’s rho coefficient (r = −0.414, *p* < 0.001) (Figure 3a), but not for volume (r= −0.042, *p* = 0.699) (Figure 3b). However, focusing on the defined individual sarcoma subgroups, SUV_max_ was not significantly higher in patients with short time survival in BS, but remained significant in STS (*p* = 0.001) and ESFT (*p* = 0.017) (Table 4 and Figure 4a). In contrast, in BS, a high tumor volume was positively correlated with a short survival period (*p* = 0.035) (Table 4 and Figure 4b). Higher TLG was only significantly correlated with short survival in ESFT but not in BS and STS (*p* =0.03) (Table 4 and Figure 4c).

### 3.4. Optimal Cutoff for Survival

**Figure 5:**
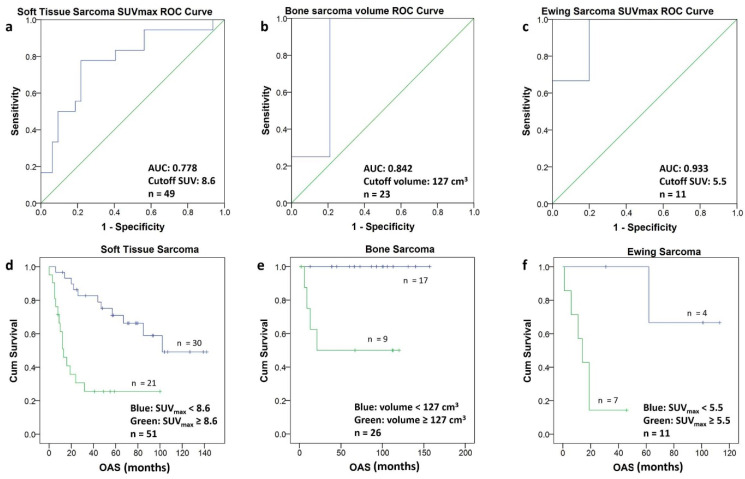
(**a**–**f**) The ROC-Curves for soft tissue sarcomas (**a**), bone sarcomas (**b**) and Ewing sarcomas (**c**) allowed to calculate a cutoff value (Youden Index) for SUV_max_ and for the volume, respectively. Using the calculated cutoff value each population was divided into two groups. The Kaplan–Meier curves of the two groups in soft tissue sarcomas (**d**) bone sarcomas (**e**) and Ewing sarcomas (**f**) are depicted. The Kaplan–Meier curves include patients with short follow-up (n = 5).

To determine the optimal cutoff value for each subgroup, the PET metric with the best discrimination between short- and long-term survival was selected, and ROC analysis yielded the following cutoffs: for ESFT an SUV_max_ of 5.5, for STS an SUV_max_ of 8.5 and for BS a volume of 127 cm^3^ (Figure 5a–c). The corresponding Kaplan–Meier graphs illustrate the significant difference in survival for the patients of the three subgroups using the above-mentioned cutoff values (Figure 5d–f).

## 4. Discussion

In concordance with the literature, we found a very wide range of survival-time for sarcoma patients who also showed considerable differences in tumor size and PET metrics. The metrics of interest were SUV_max_, representing the part of the tumor with the most activity, which can be obtained semi-automatically from the PET data, as well as volume, which can be obtained from the CT scan data manually and depending on the tumor activity and homogeneity also be estimated semi-automatically from the PET-scan data. Both SUV_max_ and volume are needed to calculate volume-based metrics, such as TLG, which is tumor volume times total SUV avidity giving information about how active a tumor is in its whole. Interestingly, we found that FDG PET-specific metrics did not correlate with survival in all the subgroups of sarcomas. While high FDG-accumulation predicted a worse outcome in ESFT (with a cutoff at SUV_max_ 5.5) and in STS (with a cutoff at SUV_max_ 8.6), the prognostic value of FDG uptake in BS could not be reproduced. In our patient population, the outcome for BS was strongly related to tumor volumes (with a cutoff at 127 cm^3^). However, due to the low patient numbers in the subgroups BS and ESFT and due to the inherently high heterogeneity in the STS subgroup comprising several histological sub-entities, we cannot rule out biases inherent to retrospective analyses.

### 4.1. Importance of Pathology and Selected Pathological Findings

Correct histopathological diagnosis of mesenchymal tumors may be challenging for the non-specialized pathologist due to the rarity of individual entities and heterogeneity of microscopical appearance. The histopathological diagnosis should therefore always be reviewed by expert pathologists at a reference center with high case load and access to specialized diagnostic methods. The expert histopathological review of the tumor tissue slides was possible for 80 out of 88 cases (91%) of the current study, with careful critical review of the pathology reports for the cases, in which slides were not available. As a result of this review, eight diagnoses have been altered and all the tumors were classified according to the modern, currently valid WHO classification of soft tissue and bone tumors [21], which we consider to be one of the strengths of our study.

Seven out of eleven patients of our ESFT group had a proven EWS gene alteration, but the group contained also three ES without a distinct mutation and one ES with a CIC-DUX4 mutation. This subpopulation of tumors may behave more aggressively than classical ES. However, the PET metrics of those patients were in line with the rest of the ESFT group, whilst the patient with the CIC-DUX4 mutation survived for 19 months (equal to the median OAS of the ESFT group with an interquartile range of 11–62 months) with this more aggressive tumor biology. Hence, we believe a greater more homogeneous group consisting of only classical ES would show an even higher correlation with SUV_max_. Another group worth mentioning are the patients with liposarcomas. In our cohort, we had a variety of different subtypes (well-differentiated, myxoid, pleomorph and dedifferentiated) with gradings ranging from G1 to G3. These different subtypes are known to have different behavior with dedifferentiated liposarcoma being much more aggressive, which was also reflected in this study with all patients who reportedly had a G1 tumor alive at the cutoff date. These two examples illustrate the importance of the correct histopathological diagnosis when interpreting our findings, but they also show the difficulties that derive from the heterogeneity of sarcomas even within one histological group, hindering a more detailed analysis for each specific subgroup.

### 4.2. Importance of Tumor Metrics

PET metrics have recently been linked to the proliferation activity of tumors as measured immunohistochemically by the nuclear expression of Ki-67, a marker for cell division, pointing to the fact that higher proliferation is associated with higher PET metrics. [22] Since most aggressive sarcomas show high proliferation rates, we could derive information about tumor biology and aggressiveness of the disease from PET-CT scans. However, there are various, sometimes contradictory results regarding PET metrics for sarcoma. A comprehensive review of the literature is given in Table 5.

**Figure 6:**
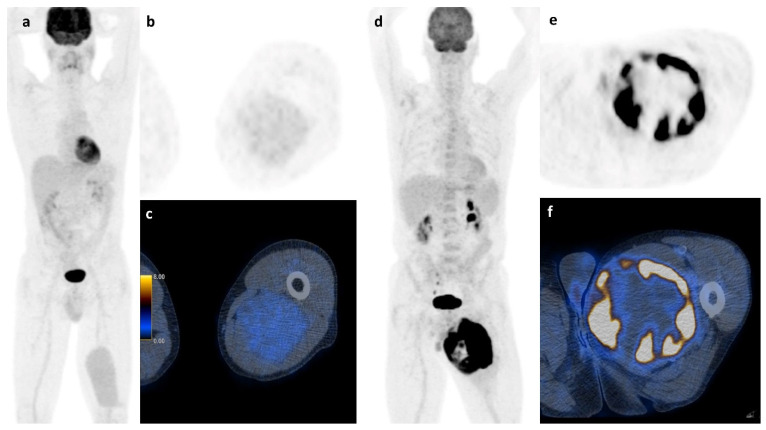
Soft tissue sarcoma: A whole body scan (**a**) and the axial images (**b**,**c**) of a 45 y.o. patient with a myxoid liposarcoma of the left thigh and a survival of at least 107 months shows low FDG-activity (2.35 SUV_max_), while the 73 y.o. patient on the right (**d**–**f**) with a pleomorphic undifferentiated sarcoma and high FDG-activity (31.60 SUV_max_) had a survival of only 12 months.

Apart from histology, simple basic metrics such as tumor volume or diameter are widely used for risk stratification in STS and are already part of guidelines and nomograms. [20,23,24] The role of neo-adjuvant or adjuvant chemotherapy in high-risk localized STS remains very controversial and new tools to stratify the risk of this patient group could further help in clinical decision-making [25,26,27,28]. FDG PET/CT was suggested to be such a potential tool that might allow an improved risk stratification. However, the assessment of FDG PET metrics throughout the literature is not uniform and ill defined, with various semiautomatic methods that lead to significant limitations of the results. For example, PET-based volume metrics using an absolute threshold of SUV_max_ (e.g., 42%) is of limited value for sarcoma assessment, since they tend to underestimate the tumor burden in highly active tumors [29]. Due to the intratumoral heterogeneity of sarcomas, we performed manual segmentation, slice by slice to ensure detailed VOI for further analysis. All segmentations were carried out by the same reader to avoid contaminating the results by interreader variability. Despite this accurate but very labor-intensive segmentation method to calculate the total tumor burden, the best predictive value for survival in our cohort of STS and ESFT was still SUV_max_ which is illustrated in Figure 6.

### 4.3. Comparison with Existing Literature

Our results are in line with part of the existing literature. [9,10,30,31] For ESFT an SUV_max_ of 5.5, for STS an SUV_max_ of 8.5 and for BS a volume of 127 cm^3^ were found to be the cutoff values for long survival. Several cut offs, e.g., 5.8 to 17.0 in ES and 6.0 to 17.7 in STS have been suggested, respectively. For BS, no volume cutoff has been reported to our knowledge. Anderson et al. reported a MTV40% of 32.6 cm^3^/mL which is not directly comparable [13]. Table 5 is giving an overview of the existing publications concerning PET metrics and survival.

In our study, volume-based metrics showed no correlation with survival in STS patients, contradicting some previous studies [13]. In synovial sarcomas, some authors found TLG and SUV_max_ to be prognostic [11]. Our cohort does not allow for further statistical analyses in synovial sarcomas alone due to low case numbers (n = 3). In rhabdomyosarcomas (RMS), SUV_max_ was described as a predictor of outcome [32]. Since our cohort only encompassed five patients who moreover showed different histological subtypes (alveolar, embryonal, and pleomorphic), we were not able to perform a meaningful statistical analysis in RMS.

Some authors found TLG to be an even better predictor of prognosis in STS than SUV_max_ [12]. There is one meta-analysis concerning the prognostic value of PET/CT metrics in STS [14], stating that not only SUV_max_ but also TLG adds prognostic information, a finding corroborated by other studies as well [33]. We could not reproduce the latter finding; however, we present further evidence that SUV_max_ could be a reliable prognostic factor in STS in general. Given the rarity of each sarcoma subgroup, we were not able to statistically analyze most of the individual subgroups. In different subgroups, different metrics may have a prognostic value, however, this needs further validation whenever large enough cohorts with the respective histology are available. In addition, small patient numbers may be one factor that leads to contradictory statements in different cohorts.

Our findings show that a higher SUV_max_ in ESFT is associated with shorter survival with a cutoff value of 5.5, which is in line with Hwang et al. who found the cutoff value to be at 5.8 [18] and others [17,19]. In our study, high TLG also correlated with short survival in ESFT patients, but less significant than SUV_max_. Volume did not correlate with survival in ESFT (*p* = 0.08) in our cohort, despite the literature suggesting such an association [7].

**Figure 7:**
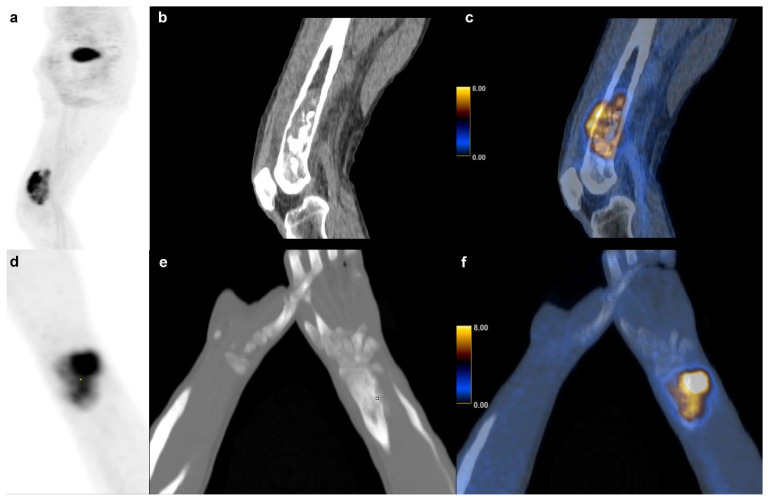
Bone sarcoma: Dedifferentiated chondrosarcoma of a 73 y.o. patient with a survival of 6 months (231.9 cm^3^) in the right distal femur (**a**) sagittal FDG PET maximum intensity projection (**b**) CT and (**c**) fused FDG PET/CT images. (**d**–**f**) Osteosarcoma in a 14 y.o. patient with a (60.1 cm^3^) of the right radius and a survival of at least 101 months.

In our cohort, SUV_max_ and other PET metrics were not significantly associated with survival in BS partly confirming the findings of some authors [9] and contradicting others who found SUV_max_ and TLG to be prognostic factors in osteosarcoma [15]. Andersen et al. also found that TLG adds prognostic value in BS, which was not the case in our study, but we found the same tendency (*p* = 0.054) [13]. Our findings showed that tumor volume itself was a significant predictor of survival in BS with a volume equal or higher than 127 cm^3^ indicative of short survival. Figure 7 shows an example of a high and low volume BS. This observation is consistent with one publication suggesting a cutoff at 150 cm^3^ for metastasis free survival [3].

We found TLG to be significantly different between the three groups with BS showing the lowest median TLG value. This could be due to the fact that the BS group consisted substantially of osteosarcomas which per definition consist of a variable volume percentage of extracellular osteoid leading to lower metabolic activity in comparison to STSs or a highly active ESFTs.

The main limitations of our study are the retrospective nature and the relatively low number of cases due to the rarity of the disease. Despite screening over 52′000 PET/CT scans not more than 88 patients with FDG PET/CT scans for primary staging could be identified. This also limited the possibility of further subanalysis regarding other important risk factors, such as age or metastatic disease. Additionally, and also owed to this diseases nature the cohort is heterogeneous in terms of the histology. This leads to very small patient groups for several sarcoma types, not allowing to investigate the entity-specific best predictive PET metric. This would be even more complex if different age groups would be considered distinct. However, we were able to analyze the ESFT as a distinct subgroup in comparison to other STSs and BSs types.

Nevertheless, a metric such as SUV_max_ for STS patients could be further used in risk stratification and to assess tumor aggressiveness, which might have therapeutic implications. Furthermore, simple PET/CT metrics might not only add information about risk stratification. It has already been a proven tool in monitoring the success of therapy using SUV_max_ decrease in sarcomas [30,34,35,36,37,38].

## 5. Conclusions

We have demonstrated that survival-time and SUV-activity are linked in STS and ESFT with a higher SUV_max_ indicating poorer survival. However, this is not the case in BS in our cohort where we found a significant association between tumor volume and survival time, irrespective of FDG accumulation. Higher tumor volume indicated poorer survival and should therefore also be considered a predictor in the group of BS. Our results need further validation given the small cohort size and more research with higher patient numbers for the various subentities has to be carried out.

## Figures and Tables

**Table 1:** Patient characteristics.

Subgroups	Bone Sarcoma (n = 26)	Soft Tissue Sarcoma (n = 51)	Ewing Sarcoma Family Tumor (n = 11)	All (n = 88)
Male/Female (n)	12/14	30/21	7/4	49/39
Age at initial PET-scan in years (median and interquartile range)	27 (16–39)	57 (36–73)	31 (19–38)	40 (25–59)
OAS in months (median and interquartile range)	70 (21–112)	44 (12–78)	19 (11–62)	46.5 (13–90)
Initial Tumor Stage (TNM)				
TX	2	12	1	15
**T1**	16	12	3	31
**T2**	7	26	7	40
**T3**	1	0	0	1
**T4**	0	1	0	1
NX	2	10	1	13
**N0**	24	39	8	71
**N1**	0	2	2	4
MX	3	9	1	13
**M0**	21	32	5	58
**M1**	2	10	5	17
Grading (as reported)				
GX	9	23	9	41
**G1**	1	8	0	9
**G2**	3	4	0	7
**G3**	13	16	2	31

**Table 2:** Detailed tumor entity information and subgroups.

Histology Subgroups			
	Bone Sarcoma	Soft Tissue Sarcoma	Ewing Sarcoma Family Tumor
Osteosarcoma (n = 17)			
- Osteoblastic	n = 6	-	-
- Chondroblastic	n = 3	-	-
- Osteo- and chondroblastic	n = 1	-	-
- High grade	n = 2	-	-
- Extrascelettal	n = 1	-	-
- Subtype unknown	n = 4	-	-
Leiomyosarcoma (n = 14)			
- non-uterine soft tissue		n = 11	-
- Of bone	n = 3		-
Ewing sarcoma family tumor (n = 11)	-	-	n = 11
Liposarcoma (n = 11)			
- Myxoid	-	n = 5	-
- Dedifferentiated	-	n = 3	-
Pleomorphic	-	n = 1	-
- Well-differentiated	-	n = 1	-
- Subtype unknown	-	n = 1	-
Angiosarcoma (n = 8)			
- Primary		n = 7	-
- Secondary		n = 1	-
Chondrosarcoma (n = 6)			
- Conventional	n = 2	-	-
- extraskeletal	-	n = 2	-
- Mesenchymal	n = 1	-	-
- dedifferentiated	n = 1	-	-
Rhabdomyosarcoma (n = 5)			
- alveolar	-	n = 3	-
- embryonal	-	n = 1	-
- pleomorphic	-	n = 1	-
Undifferentiated pleomorphic sarcoma (n = 4)		n = 4	
Synovial sarcoma (n = 3)	-	n = 3	-
Undifferentiated high-grade pleomorphic sarcoma of bone	n = 2	-	-
Myxofibrosarcoma (n = 2)	-	n = 2	-
Intimal sarcoma	-	n = 1	-
Sclerosing Epithelioid Fibrosarcoma	-	n = 1	-
Low-grade fibromyxoid sarcoma (Evans Tumor)	-	n = 1	-
Malignant peripheral nerve sheath tumor	-	n = 1	-
Follicular dendritic cell sarcoma	-	n = 1	-
Total (n = 88)	n = 26	n = 51	n = 11

**Table 3:** Metrics between the 3 subgroups.

	BS (n = 26)	STS (n = 51)	ESFT (n = 11)
SUV_avg_	3.2	3.6	3.3
Mean (range)	(2.4/0.5)	(2.4/0.3)	(1.8/0.5)
SUV_min_	0.6	1.1	0.7
Mean (range)	(0.3/0.1)	(1.1/0.2)	(0.4/0.1)
SUV_max_	9.6	8.7	8.1
Mean (range)	(7.7/1.5)	(6.2/0.9)	(4.8/1.4)
TLG	465.7	808.0	2727.7
Mean (range)	(558.0/109.4)	(1147.0/202.6)	(5268.7/1588)
Volume [cm^3^]	145.1 (181.2/36.2)	359.1 (476.0/66.7)	354.4 (430.5/136.1)
HU_avg_	189.8 (93.8/18.8)	34.0 (41.3/5.8)	89.7 (109.3/34.6)

**Table 4:** PET and volume metrics and survival period ^a^.

	SUV_max_	TLG	Volume [cm^3^]
***Soft tissue sarcoma***			
short survival (n = 18)	12.5 (8.8–16.0)	291 (124–871)	61 (26–398)
long survival (n = 31)	5.5 (3.3–7.2)	329 (75–879)	142 (28–405)
*p*-value *	0.001	0.642	0.613
***Ewing sarcoma***			
short survival (n = 6)	12.1 (7.6–14.7)	2594 (827–3719)	457 (262–1146)
long survival (n = 5)	3.7 (3.5–5.5)	85 (46–245)	29 (27–149)
*p*-value	0.017	0.030	0.15
***Bone sarcoma***			
short survival (n = 4)	7.8 (7.1–9.0)	656 (487–1022)	217 (186–349)
long survival (n = 19)	7.0 (4.4–11.6)	165 (42–403)	60 (22–104)
*p*-value	0.557	0.054	0.035

^a^: Values expressed as median and interquartile range, * *p*-values of two-tailed Wilcoxon test between long and short survival groups, a *p*-value < 0.05 is considered significant.

**Table 5:** Literature summary ^a^.

Author	Histology	No of Pat.	PET metric Corr. with Survival	PET metric Not Corr. with Survival	Cut off	AUC
Hong 2014 [10]	STS (but including ES (8))	55	SUV_max(+avg)_	MTV + TLG	-	-
Andersen * 2015 [9]	STS (55)	55	SUV_max_ T/B TLG MTV40%		17.7 7.2 265.6 g 25.0 mL	0.797 0.787 0.780 0.694
Andersen * 2015 [13]	BS (37 including 6 ES)	37	TLG MTV40%	SUV_max_ T/B	11.6 8.0 149.4 g 32.6 mL	0.630 0.593 0.773 0.727
Schuetze 2005 [30]	STS	47	SUV_max_ correlating with DFS und MFS but not OAS	(SUV_max_ (OS))	Predefined > 6	-
Skamene 2014 [31]	STS, BS and ES	81, 23 and 16	SUV_max_		10.3	-
Chang 2015 [11]	STS (Synovial sarcoma)	20	SUV_max_ MTV2.5abs TLG		6.1 166.2 mL 691.7 g	- - -
Casey 2014 [32]	STS (Rhabdomyosarcoma)	107	SUV_max_		(predefined 6.0)/9.5	-
Choi 2013 [12]	STS	76	TLG SUV_max_ MTV40%		250 g 6.0 40 cm^3^	0.833 0.771 0.667
Ha 2016 [33]	STS (head and neck)	36	SUV_max_ SUV_peak_ MTV TLG	Tu Vol	7.0 5.0 20 mL 150.0 g 15.0 mL	0.779 0.753 0.716 0.739 0.682
Hwang 2016 [31]	ES	34	SUV_max_		5.8	-
Salem 2015 [19]	ES	28	SUV_max_		11.6	-
Jamet 2017 [17]	ES	32	SUV_max_ SUV_peak_	MTV TLG	17.0 12.5 - -	- - - -
Costelloe 2009 [15]	BS (Osteosarcoma)	31	SUV_max_ (only PFS not OS) TLG	(SUV_max_ (OS))	- -	- -

^a^: T/B = Tumor Background ratio, MFS = Metastatic free survival, PFS = progression free survival und OS =overall survival). * Two publications with the same population merged.

## Data Availability

Patient imaging was done in the scope of routine clinical diagnostic studies, and the raw data are stored in the hospital archiving system at the Zurich University Hospital, Zurich, Switzerland.
